# A randomised clinical trial to evaluate the safety, fit, comfort of a novel N95 mask in children

**DOI:** 10.1038/s41598-019-55451-w

**Published:** 2019-12-12

**Authors:** Daniel Yam Thiam Goh, Meng Wai Mun, Wei Liang Jerome Lee, Oon Hoe Teoh, Dimple D. Rajgor

**Affiliations:** 1 Department of Paediatrics, Yong Loo Lin School of Medicine, National University of Singapore, Singapore, Singapore; 2 Khoo Teck Puat-National University Children’s Medical Institute, National University Health System, Singapore, Singapore; 3 Innosparks Pte Ltd, Singapore, Singapore; 4 Department of Paediatrics, KK Women’s and Children’s Hospital, Singapore, Singapore

**Keywords:** Paediatric research, Paediatric research

## Abstract

Children are more vulnerable to the risks of air pollution, including susceptibility to acquiring chronic diseases in their developing lungs. Despite these, there are no specific masks designed for and tested in children that are available to protect our young from the common particulate air pollutants today. We evaluated safety, fit and comfort of a specially designed paediatric N95 mask with an optional micro ventilator (micro fan, MF) in healthy children aged 7–14 years, in a randomized, two-period crossover design. The subjects’ cardiorespiratory physiological measurements were assessed in different states of physical activity under different interventions (mask without and with MF). A total of 106 subjects were recruited between July-August 2016. The use of the mask without MF increased the End-Tidal CO_2_ (ETCO_2_) and Fractional concentration of Inspired CO_2_ (FICO_2_) at rest and on mild exertion, as expected. The use of the mask with MF brought FICO_2_ levels comparably closer to baseline levels without the mask for both activities. The mask, with or without the MF, was found to be well fitting, comfortable and safe for use in children at rest and on mild exertion. The N95 mask tested offers a promising start for more studies in the paediatric population.

## Introduction

Air pollution, such as haze or smog, is an evolving and increasingly significant problem around the world. A wide range of hazards of chronic air pollution in children include nocturnal cough, asthma, poor performance in neurobehavioral function, negative impact in cognitive development and harmful effects on brain development^1–5^. In particular, children represent a vulnerable segment of any population. They carry more risks of long term exposure to pollution over the course of their lives and are susceptible to acquiring chronic diseases in their developing lungs^6–11^. Use of masks and respirators can offer protection against air pollutants. However, the commercially available disposable particulate respirators, typically certified for surgical (which may only offer some barrier against larger particles) and occupational use, are mainly designed for and studied in adults. The test standards are specified according to adult breathing conditions and fit. There are to date, no masks designed for and evaluated in children.

Several studies have investigated the effectiveness, safety, fit and comfort of different types of masks^12–15^. However these studies were done only in adult populations. Even alternatives such as cloth masks have been tested only in adult populations^16^. In another study, the facemasks for paediatric use (FPU, the masks that are not specifically designed for paediatric use but are the existing mask that may be used for children during emergency situation like that of airborne disease outbreaks) were tested mainly to evaluate the leakage associated with donning the FPU^17^. This study did show superiority of FPU in comparison to surgical masks in certain aspects, however, the study was not performed in children. One study was performed in children to evaluate the redesigned open system face mask. However, the objective of this study was to evaluate the mask for monitoring P_ETCO2_ during sedation in clinical practice and the children in this study donned the mask only for 30 sec.^18^. These and other similar studies merely point to the fact that there are no masks that are specifically designed and tested in children such that they can be prescribed for paediatric use in the setting of daily routine activities.

In our study, the N95 masks were specifically designed for use in children and they were evaluated in children under conditions of routine daily activities. They are the first to be paired with a micro ventilator (micro fan, MF), designed to reduce the accumulation of exhaled carbon dioxide (CO_2_), heat and humidity in the dead space of the respirator by venting out the expired air. This is to enhance the comfort and experience in wearing the mask. This novel mask comprises a valved, disposable respirator which has the option of being paired with a reusable lithium battery powered MF.

This study was designed to evaluate the safety, fit and comfort of the novel disposable N95-class particulate respirator with and without MF, in healthy children aged 7 to 14 years without any underlying medical condition. The masks were specifically developed for the school going children aged 7 to 14 years since the children in this age group are largely school-going and would be involved in routine activities which would expose them to outdoor environment pollutants especially during seasonal or periodic haze. Children below 7 years of age are more likely to be confined to indoor environments while children above 14 years can use the masks certified for adult use.

## Methods

### Trial design

This was a randomised, two-period crossover study in which the subjects were randomly assigned to one of the two sequences of the interventions (Trial Registration: ClinicalTrials.gov, NCT03252574, August 17, 2017). The study was approved by the National Healthcare Group Domain Specific Review Board (NHG DSRB Reference: 2015/01059) and the informed consent was obtained from the parent or legal guardian as well as consent/assent as appropriate from children, prior to enrolment in the study for their study participation. All methods were performed in accordance with the relevant guidelines and regulations.

### Randomization and blinding

Being a cross over study, each study subject served as their own control for comparison and received all interventions, eliminating the influence of selection bias. To avoid influence of the sequence in which the interventions were given, the study subjects were manually randomized to one of the two sequences of interventions and then crossed over. For example, the first eligible participant was asked to go to station A-B, the second B-A, the third A-B, the fourth B-A and so on.

Given the nature of the interventions used; without mask, with mask and, with mask and MF, it was not possible to blind the investigators. However, all the assessments were objectively measured by the sensors built in the device itself, thus avoiding the assessment bias associated with un-blinded investigators.

### Participants

Children aged 7 to 14 years old, from two schools in Singapore, were invited to participate in the study by sending an invitation letter to the parents. The selection of the two schools was based on the availability of the participants within the desired age range (7 to 14 years) and the schools agreeing to participate in the study. These were regular schools in the community and would be fairly representative of the regular school-going child in Singapore. The subjects were randomly selected from the pool of eligible children meeting the inclusion-exclusion criteria listed below.

### Inclusion criteria

The subjects meeting both inclusion criteria listed below were eligible to enrol in the study.

1. Aged between 7 and 14 years of age (inclusive).
2. The parents or legal guardians of the subjects must provide their consent to take part in the study.

### Exclusion criteria

The subjects fulfilling any of the exclusion criteria listed below were excluded.

1. any known cardiorespiratory conditions (including but not limited to the following: asthma, bronchitis, cystic fibrosis, congenital heart disease, emphysema)
2. any known medical conditions that may be exacerbated by strenuous physical activity, including but not limited to the following: exercise-induced asthma, lower respiratory infections (including pneumonia, bronchitis) in the past 2 weeks, anxiety disorders, diabetes, hypertension, or epilepsy/seizure disorders
3. any physical disability from medical, orthopaedic or neuromuscular disorders
4. have an acute upper respiratory tract infection or symptomatic rhinitis (i.e. blocked nasal passages, runny nose or significant sneezing) on the day of the study
5. may have conditions or abnormalities that may compromise the integrity of the mask fit (e.g. those with excessive facial hair or craniofacial abnormalities)

An on-site doctor and a nurse administered the screening questionnaire to assess the subjects’ pre-investigation condition. Each participant underwent a mask fit test to determine the right mask size for the face, including testing for leakage. Participants were excluded if he/she was unable to be fitted with any of the mask sizes as determined by the mask fit and leak tests.

### Mask fit test

The test involved performing the following exercises in sequence: normal breathing, deep breathing, turning head from side to side, up and down, speaking and bending exercise. The equipment used was the TSI Portacount 8038 respirator fit tester. A good or ‘pass’ fit was based on the fit factor as per the respirator certification EN 149:2001 + A1:2009 FFP2 standards.

### Interventions

**Figure 1:**
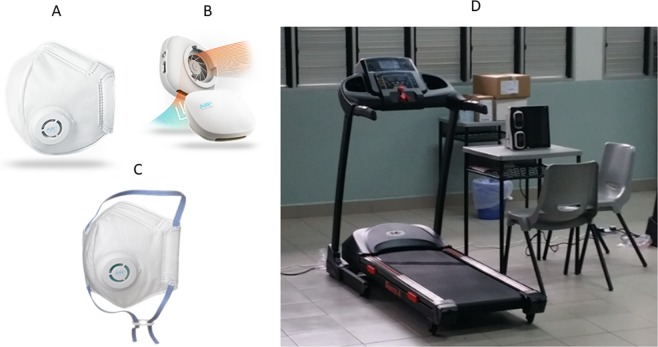
Illustration of the (**A**) disposable N95-class particulate respirator that is able to be paired with the (**B**) novel, reusable micro fan (**C**) AIR^+^ Smart Mask (**D**) Experimental set up.

**Figure 2:**
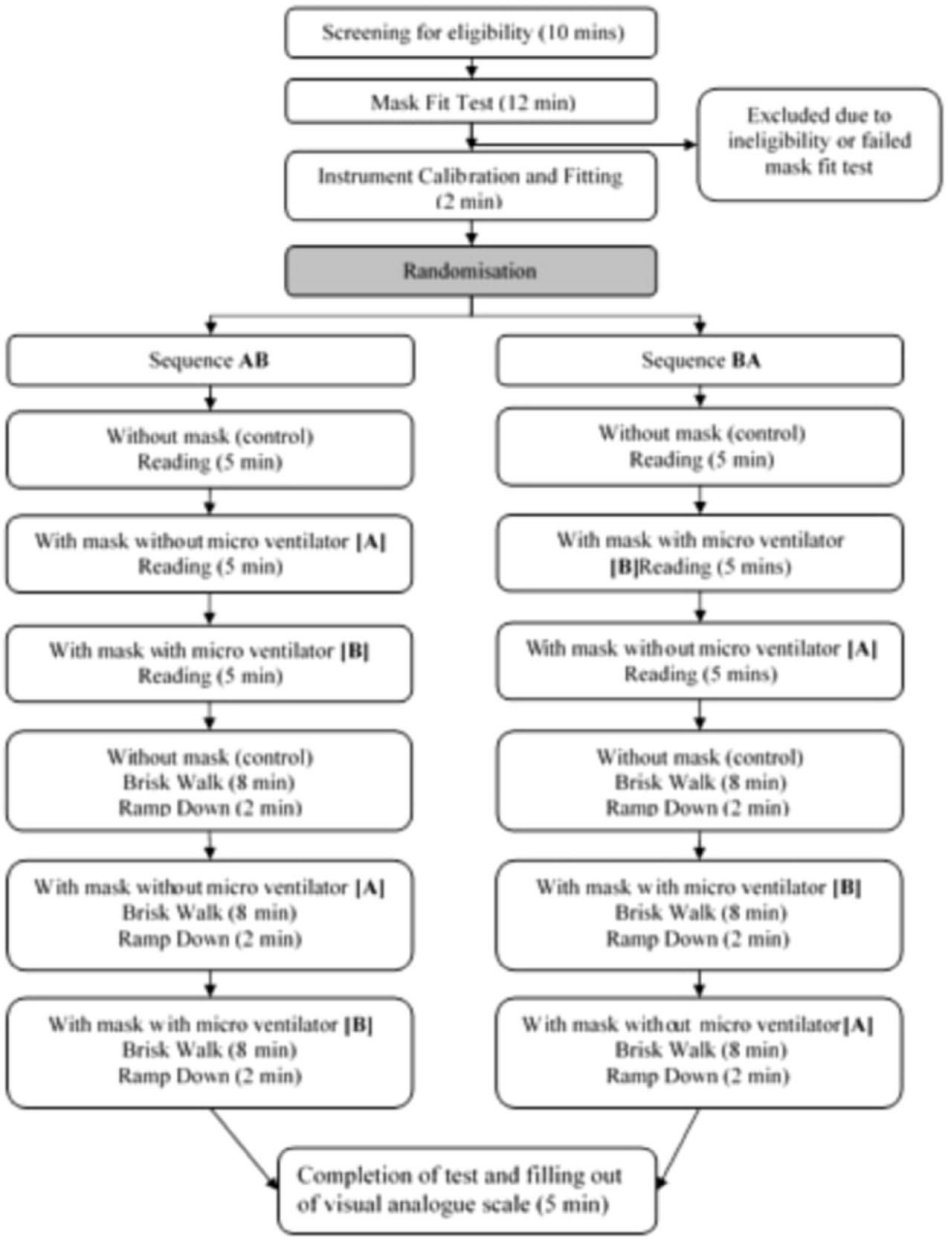
Process flow of the study design.

The mask and MF used in the study, as well as the experimental set up are depicted in Fig. 1. The subjects were randomly assigned by sequential allocation into one of two sequences, Sequence AB (No mask [Control], followed by AIR^+^ Smart Mask only [A], then AIR^+^ Smart Mask with MF [B]) or Sequence BA (No mask [Control], followed by AIR^+^ Smart Mask with MF [B], then AIR^+^ Smart Mask only [A]) (Fig. 2).

The impact on subjects’ physiological measurements following different interventions (A and B) was assessed in different states of common physical activities such as at rest (reading) and on mild exertion (brisk-walking on the treadmill) to represent the daily routine activities for children such as reading in school or walking a short distance.

Briefly, each participant randomized to Sequence AB, was asked first to read, seated for 5 minutes during which baseline readings for all parameters were measured (Control). The participant then donned the mask without the MF for another 5 minutes. Finally, the MF was attached to the valve of the mask and the participant read for a final 5 minutes to complete the rest phase of the study.

The above procedure was repeated for the physical activity of mild exertion when the subjects performed brisk walking on the treadmill instead of reading. The brisk walk was targeted at reaching their target heart rate (HR) at mild exertion.

Mild exertion was achieved through the use of the treadmill at speeds that represent realistic activity levels during play. As the subjects were of varying ages and heights, with correspondingly different levels of physiological reserves, it may not be feasible to assign a specific treadmill speed that would indicate a uniform level of mild exertion. Based on the known normal resting HR of healthy children ages 7–14 years (70 to 100 beats per minute), this study aimed to keep the actual speed of the treadmill flexible and primarily target a heart rate zone of 50–60% of the subjects calculated maximum HR. The rationale for choosing this target is based on studies in adults where achieving a target HR zone of 50–60% of maximum HR would yield an exercise intensity of about 40% of maximal oxygen consumption (VO2max), which is a good indicator of aerobic endurance. For adults, the anaerobic threshold is about 50–60% of VO2max. There is no literature on VO2max for children, hence, to be conservative, we have referenced adult threshold and taken 40% of VO2max as children’s normal anaerobic threshold. As such, to attain a stable ETCO2 during aerobic exercise and before the subject crosses the anaerobic threshold, we aim to keep to 50–60% of predicted maximal HR.

The most common method to estimate maximum HR within the exercise community is to use the 220 – age formula^19,20^. This study proposed a more conservative assumption of a maximum HR of 194 bpm, and a target HR zone to be achieved on the treadmill of between 100–120 bpm. As an additional safety measure, subjects were encouraged to talk comfortably while on the treadmill. This would indicate that the activity level is appropriate. The child was led to a target HR before 3 minutes of steady state data was taken. Given the physiological differences between different children, as well as their varying levels of fitness, different times were required to attain the requisite HR for the test.

Hence, in summary, we aimed at achieving mild physical activity comparable to a brisk walk and chose a target HR of approximately 50 to 60% of the calculated max HR as standardization of the level of physical activity.

At the end, the participants were asked to slow their walk to a leisurely pace on the treadmill for 2 minutes before the treadmill was stopped and their heart rates returned to the resting level. Two standard treadmills were used to achieve the target heart rates of between 100 to 120 bpm to simulate mild exertion^21^. The test was carried out in an open, naturally ventilated classroom in each of the two schools. The approximate ambient conditions in the classrooms were as follows: temperature range of 26 to 32 °C, relative humidity between 50–80%, and wind speed of <1.5 m/s (with a ceiling fan at medium speed).

For subjects randomized to Sequence BA, all of the above activities were repeated by reversing the sequence of MF use, B followed by A (Fig. 2).

Given the variability of physiological measurements between children, a randomised crossover design allowed for a more precise comparison of different interventions, and required a smaller number of subjects than a between-subjects study.

### Outcomes and assessments

The primary outcome of the study was the end-tidal carbon dioxide (ETCO_2_) levels to assess the safety of the mask in terms of potential carbon dioxide retention. The mask fit was assessed using the mask fit test. The comfort level of the masks worn was assessed on a visual analogue scale (VAS). Despite being a subjective assessment tool, it has the advantage of ease of rating specifically when used by children. In addition, the VAS has been shown to be a valid tool corroborating well with detailed questionnaires when used as a single question rating^22^.

Single-use nasal cannulas were used in conjunction with Nomoline™ Adapters connected to a Masimo Root^®^ Patient Monitoring System with an ISA™ CO_2_ module to log the ETCO_2_, fractional inspired carbon dioxide (FICO_2_) and respiratory rate (RR) information.

M-LNC Pediatric adhesive sensors were worn by the subjects and the Masimo Radius-7™ was used to log oxygen saturation (SpO_2_) and pulse rate (PR).

### Sample size

It was anticipated that a child at rest will have a mean ETCO_2_ of 40 mmHg with a standard deviation of approximately 8mmHg^23^. Based on the proposed safety threshold ETCO_2_ level of below 50 mmHg^24,25^, an increase of ≤10 mmHg after the subject wears the mask with or without the MF would indicate non-inferiority. We elected to adopt a more stringent tolerance level of less than 5 mmHg increase in ETCO_2_ in this study.

Assuming a one-sided test size of 5% and one-sided power of 80%, at least 80 subjects were required. To account for the children who may withdraw during the study, a total of 100 children were proposed to be recruited, distributed across the age groups.

### Statistical analysis

The 95% upper confidence limit of the increase in ETCO_2_, the primary outcome of the study, after wearing a mask without or with MF was computed. The mean (SD) values of the primary outcome and other physiological parameters (RR, HR, SpO_2,_ FICO_2_) obtained at rest and at mild exertion were presented descriptively. The % increase or decrease in the outcome variables over the control group values was computed to understand the physiological effect of wearing the mask with and without MF. The comfort of the mask from the VAS data was presented descriptively.

### Ethics approval and consent to participate

The study was approved by the National Healthcare Group Domain Specific Review Board (NHG DSRB Reference: 2015/01059) and the consent was obtained from the parent or legal guardian prior to enrolment in the study.

## Results

### Participant demographics

A total of 106 subjects were recruited in the study between July and August 2016. There were 59 (55.6%) boys indicating equal distribution of the gender in the study population. The mean (SD) weight, height and BMI of the participants were, 41.4 (15.3) kg, 144.6 (17.4) cm and 19.1 (5.4) kg/m^2^ respectively. The physiological parameters are summarized in Table 1. All subjects recruited were successfully fitted with the mask and all passed the mask fit test. No participants had to be rejected because of inability to fit a mask.

**Table 1:** Physiological parameters of the study subjects.

Physiological parameters (N = 106)	Without mask (Control)	With mask only (A)	With mask and with micro fan (B)
**Mean (SD) ETCO**_**2**_**, mmHg**			
At rest (Reading)	30.9(3.37)	34.3(3.32)	32.2(3.27)
On mild exertion (Brisk walking)	28.2(2.8)	32.0(2.8)	30.6 (3.1)
**Mean (SD) FICO**_**2**_**, mmHg**			
At rest (Reading)	8.2 (1.94)	10.7 (2.61)	7.8 (2.15)
On mild exertion (Brisk walking)	9.9 (1.69)	12.1 (2.47)	10.8 (2.33)
**Mean (SD) RR, bpm**			
At rest (Reading)	18.0 (3.21)	17.4 (3.57)	16.8 (3.33)
On mild exertion (Brisk walking)	23.5 (3.53)	23.2 (3.73)	23.2 (3.88)
**Mean (SD) HR/bpm**			
At rest (Reading)	89.6 (11.31)	90.7(11.37)	89.2 (11.27)
On mild exertion (Brisk walking)	108.4 (9.84)	110.2(7.73)	109.5 (7.78)
**Mean (SD) SpO**_**2**_**/%**			
At rest (Reading)	99.6 (0.52)	99.5 (0.51)	99.6 (0.54)
On mild exertion (Brisk walking)	99.2 (0.81)	99.2 (0.65)	99.2 (0.78)

ETCO_2_ = end-tidal carbon dioxide, FICO_2_ = fractional inspired carbon dioxide, RR = respiratory rate, SpO_2_ = oxygen saturation, HR = Heart rate.

### Outcomes and assessments

#### Safety of the mask

ETCO_2_. All the children wearing the mask alone had ETCO_2_ values below 50 mmHg. The highest ETCO_2_ value recorded was 42 mmHg.

The mean ETCO_2_ values at rest were 30.9 mmHg, 34.3 mmHg and 32.2 mmHg for the subjects without mask, with mask, and with mask and MF respectively. The corresponding mean values at mild exertion were 28.2 mmHg, 32.9 mmHg and 30.6 mmHg for the subjects without mask, with mask, and with mask and MF respectively. Whether at rest or on mild exertion, the ETCO_2_ values were higher when the mask was worn but the values decreased, approaching the control values when the mask was worn with the MF.

**Figure 3:**
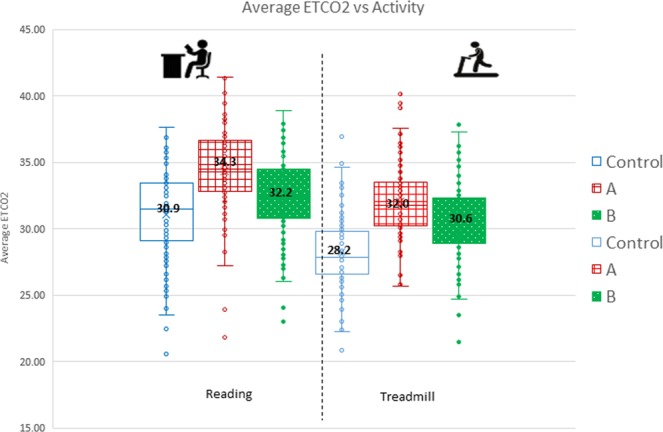
Mean ETCO_2_ values at rest and on mild exertion for Control, Intervention (**A**) and Intervention (**B**).

The mean ETCO_2_ values on mild exertion were observed to be consistently about 2 mmHg lower in comparison to when at rest. This is attributed to CO_2_ levels being lower from increased minute ventilation as a result of exercise; as respiratory rate and tidal volume increased, resulting in the decrease in CO_2_ levels (Fig. 3).

FICO_2_. The mean FICO_2_ values at rest were 8.2 mmHg, 10.7 mmHg and 7.8 mmHg for the participants without mask, with mask, and with mask and MF respectively. The corresponding mean values at mild exertion were 9.9 mmHg, 12.1 mmHg and 10.8 mmHg for the subjects without mask, with mask, and with mask and MF respectively.

**Figure 4:**
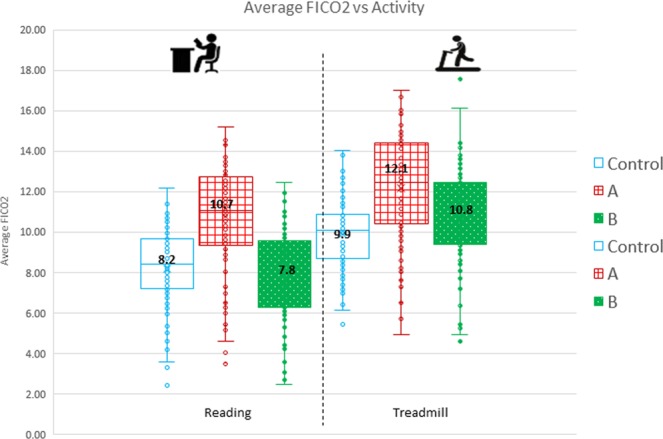
Mean FICO_2_ values at rest and on mild exertion for Control, Intervention (**A**) and Intervention (**B**).

Similar to ETCO_2_, the FICO_2_ values also appeared to increase when the mask was worn. However, when the MF was used, it decreased FICO_2_ levels towards the control level, approximating to the baseline levels seen when not wearing a mask (Fig. 4).

**Figure 5:**
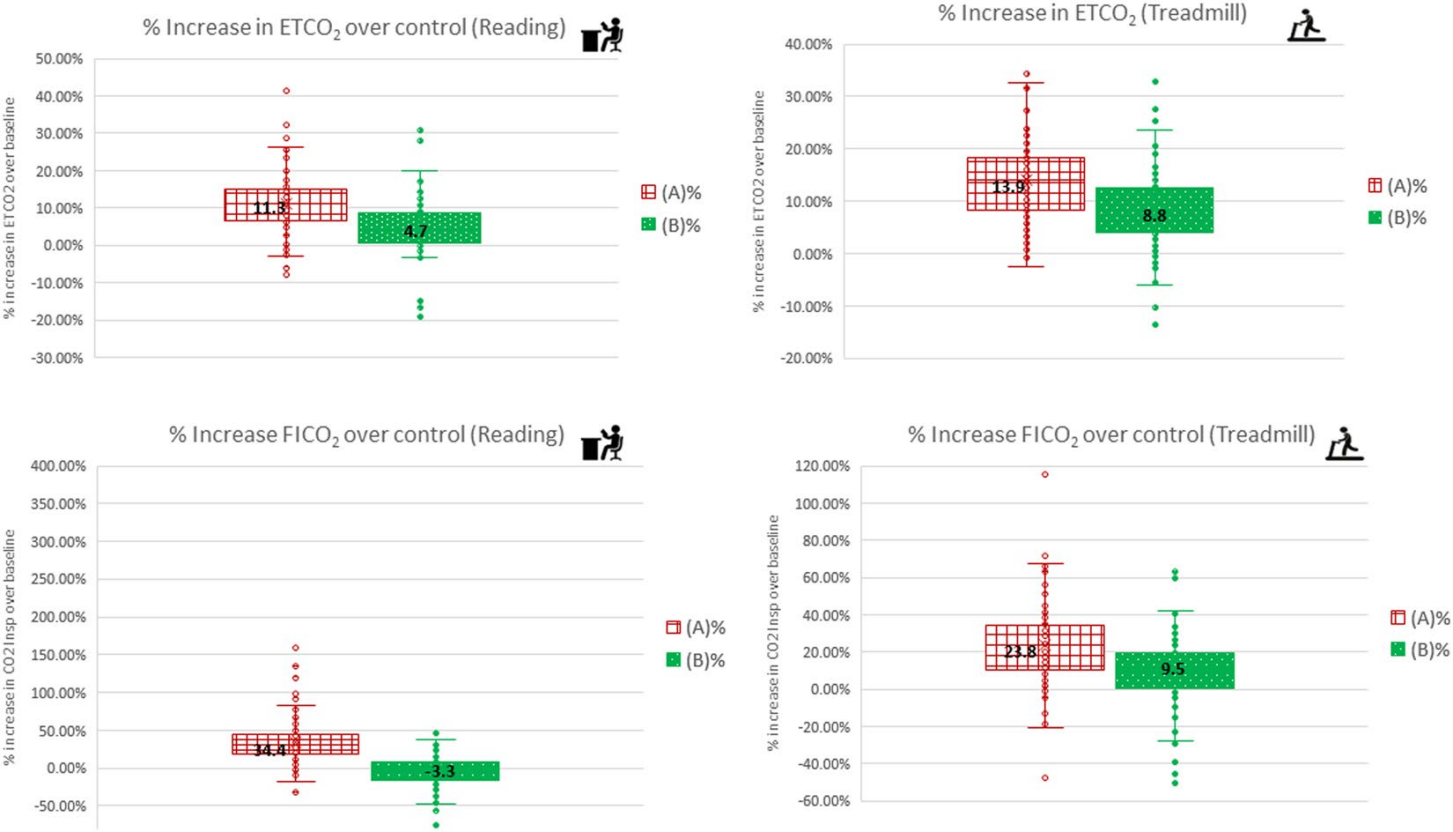
Percentage change in ETCO_2_ and FICO_2_ for Intervention (**A**) and Intervention (**B**).

Change in ETCO_2_ and FICO_2_ values. At rest, the % increase over control for ETCO_2_ and FICO_2_ when mask was worn was 13.9% and 23.9% respectively. However, this increase was minimal when the mask was worn with the MF, 8.8% and 9.5% for ETCO_2_ and FICO_2_ respectively (Fig. 5).

Similarly, at mild exertion, the % increase over control for ETCO_2_ and FICO_2_ when the mask was worn was 11.3% and 34.4% respectively. However, this increase was minimal or even better than control (i.e. not wearing a mask) when the mask was worn with the MF, 4.7% and −3.3% for ETCO_2_ and FICO_2_ respectively (Fig. 5).

The values of other physiological parameters (RR, HR, SpO_2_) obtained in the study are presented in the Tables 1 and 2.

**Table 2:** Comparison of the mean ETCO_2_, FICO_2_ and other physiological parameters for subjects indicating experiencing mild breathing difficulty on VAS (7) vs the rest of the participants (99).

Physiological parameters	Without mask (Control)		With mask only (A)		With mask and with micro fan (B)	
	7 subjects	99 subjects	7 subjects	99 subjects	7 subjects	99 subjects
**Mean (SD) ETCO**_**2**_**, mmHg**						
At rest (Reading)	29.1 (2.89)	30.9(3.4)	32.9 (3.18)	34.3 (3.3)	30.1 (4.16)	32.2 (3.3)
On mild exertion (Brisk walking on treadmill)	27.1 (2.02)	28.2 (2.8)	31.2 (1.61)	32.0(2.8)	29.8 (1.27)	30.6 (3.1)
**Mean (SD) FICO**_**2**_**, mmHg**						
At rest (Reading)	7.9 (2.11)	8.2 (1.9)	9.8 (2.58)	10.7 (2.6)	7.7 (1.52)	7.8 (2.1)
On mild exertion (Brisk walking on treadmill)	9.4 (1.46)	9.9 (1.7)	11.7 (2.05)	12.1 (2.5)	10.6 (2.41)	10.8 (2.3)
**Mean (SD) RR, bpm**						
At rest (Reading)	17.6 (3.15)	18.0 (3.2)	16.7 (3.67)	17.4 (3.6)	16.2 (3.47)	16.8 (3.3)
On mild exertion (Brisk walking on treadmill)	23.4(2.79)	23.5 (3.5)	23.5 (4.28)	23.2 (3.7)	23.4 (4.04)	23.2 (3.9)
**Mean (SD) HR /bpm**						
At rest (Reading)	91.8 (10.67)	89.6 (11.3)	92.3(11.91)	90.7 (11.4)	91.1(12.77)	89.2 (11.3)
On mild exertion (Brisk walking on treadmill)	108.7(11.66)	108.4 (9.8)	109.4(9.82)	110.2 (7.7)	110.5(7.63)	109.5 (7.8)
**Mean (SD) SpO**_**2**_**/%**						
At rest (Reading)	99.1 (0.75)	99.6 (0.5)	99.0 (0.77)	99.5 (0.5)	99.1 (0.77)	99.6 (0.5)
On mild exertion (Brisk walking on treadmill)	99.0 (0.61)	99.2 (0.8)	99.1 (0.45)	99.2 (0.6)	99.1 (0.49)	99.2 (0.8)

ETCO_2_ = end-tidal carbon dioxide, FICO_2_ = fractional inspired carbon dioxide, RR = respiratory rate, SpO_2_ = oxygen saturation, HR = Heart rate.

Mask fit. The masks used were found to fit all the children tested in the study.

Comfort of mask worn and physiological parameters for children rating mild breathing difficulty. The VAS showed that 93% of the children experienced no breathing difficulty when using the mask while 7% (4 males and 3 female subjects) indicated that they experienced mild breathing difficulty. The mean values of ETCO_2_, FICO_2_ and other physiological parameters for these 7% subjects were comparable to the rest of the study population and were well within acceptable ranges (Table 2) indicating that none of these subjects had any clinically significant respiratory compromise despite their reported subjective discomfort.

**Figure 6:**
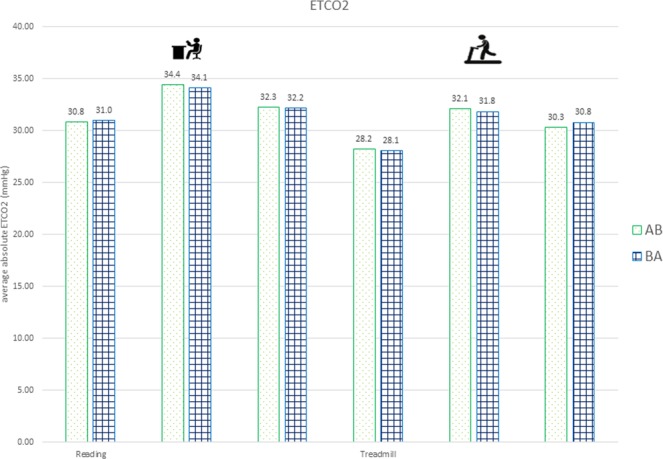
Sequence AB and BA did not have noticeable impact on ETCO_2_ results.

Impact of the randomisation sequence: The readings were independent of the sequence of the intervention (Fig. 6).

## Discussion

In our study, the physiological parameters; ETCO_2_, FICO_2_, RR, HR, SpO_2,_ were well within the acceptable range, the masks used were found to fit all the children tested in the study and majority of the children did not experience any difficulty in breathing while wearing the masks indicating good comfort. The two physical activities included in the study, reading and brisk walking on treadmill, represent the daily routine activities for children such as reading in school or walking a short distance (for example, walking to a bus stop to catch bus) indicating that the masks can be safely and effectively used during such routine activities without any respiratory compromise.

This is the first study done to assess the specially designed N95 mask for paediatric participants hence there is no comparable data to reference. Comparisons with studies in adult subjects would not be relevant in view of the different physiological setting and the aims of the studies would be different. Nevertheless, with the use of data from the control group included in the current study, the results are self-explanatory and provide evidence of the safety, fit and comfort of the mask designed for and applied in paediatric subjects.

Although safety and efficacy are clinically important, the fit and comfort determine the compliance to and efficacy of the mask. Masks with inadequate fit would not offer optimal protection in children against hazardous particulate matter (PM) including PM 2.5, the main air pollutant found in transboundary smoke and haze. Poor comfort is repeatedly brought up as the main hindrance for compliance in studies done in adult populations^26–29^. In adults, despite poor fit and comfort, compliance can still be achieved through the understanding of the benefits and need, but this may not be the case in children. Hence, the fit and comfort are essential determinants for the acceptance of the mask from the wearer’s perspective and since they also contribute to its effectiveness, they are especially critical in children.

Although more comfortable, surgical masks (SM) and also medical masks (MM), tested in various studies have been shown to perform poorly as protection against airborne and particulate infection compared to N95 masks^30–34^. Hence the masks under evaluation in this study are N95 masks that are specifically designed for children.

Generally, when masks are worn, they increase the resistance of breathing resulting in increase in CO_2_ in the dead space of the mask. As a result, the wearer encounters increased work of breathing needing more effort, causing discomfort and fatigue. The MF used in the study was found to effectively decrease FICO_2_ levels, bring them closer to the control level, approximating to not wearing a mask consistent with the results in computational experiments^35^.

The provision of the MF as an optional device for better air exchange and reducing heat and moisture build up, when needed, is also one of the recommendations of the Project BREATHE^28^. Earlier simulation experiments done in adult subjects have demonstrated that the introduction of the fan to the N95 mask is a feasible option to reduce the CO_2_ accumulation in the dead space and also to control the temperature^36^.

Vaccination and antiviral agents as preventive and therapeutic measures are important in disease control, however the prevention of disease transmission plays a vital part in preventive medicine and infectious disease control^37^. Studies have established the benefits of masks as a non-pharmaceutical modality that serves as a barrier between the virus and the subjects thus breaking the transmission of virus into human host^38,39^. In fact, during pandemics or disease outbreaks, vaccines may not be available or able to effect its benefits immediately. In such situations, the use of effective masks to prevent the spread of pathogens remains the one and only useful intervention. Children are often a more vulnerable group to infectious disease complications. At the same time they may be more capable of spreading disease as they may shed viruses for longer periods of time compared to adults^40,41^. The use of effective masks can therefore be even more impactful in children and its effectiveness (including comfort and fit) are especially pertinent in children. Be it in disease outbreaks or environmental disasters, protecting self or protecting others from self, masks remain the mainstay of respiratory disease protection.

The study has a few limitations. In our study, the children wore the mask for short durations of time, just about 5 minutes. Since this was the first study done in children to evaluate the safety and efficacy of novel N95 masks, we kept it very simple with minimum evaluations and shorter exposure time to ascertain the compliance. We believe, the study still provides the basis for future studies that can be designed to evaluate the impact of wearing the mask or mask with MF, for longer durations. Although, assessing the mask fit in children from other geographic regions due to variability prevailing in the anthropometric characteristics may be suggested, given the ethnic mix of population residing in Singapore, as well the preliminary data available for the Sellion to Menton Measurements, we believe, the mask will reasonably fit children from other parts of the world too. The preliminary data on the facial screening and measurement for the Sellion to Menton Measurements of Singapore children aged 7 to 14 years (Mean [range] of 9.63 [7.43–15.96] cm) suggests that the measurements compared well with the data of children of similar age from US (Mean [range] 10.16 [7.59–12.29] cm)^42^.

Multiple *in-vitro* and animal studies have already been undertaken to evaluate the biocompatibility of the mask used in the study. The results of these studies have shown no delayed hypersensitivity on the skin of the tested Guinea pigs, causing no intracutaneous irritation in New Zealand White Rabbit, showing no cytotoxic effect in terms of cell lysis or reduction of cell growth. The masks have shown the bacterial and viral filtration efficiency of >99% and synthetic blood penetration resistance test passing quality standards of Per ASTM F1862 and ISO 22609.

With the above data, the findings of this preliminary study done in healthy children opens up avenues for future research using the mask tested in this study. Use of these masks in children with cardiopulmonary disease including asthma, respiratory diseases, as well as testing and evaluating the masks in vulnerable children for quantitative risk assessment will have to be undertaken to understand and expand the potential use of the mask. Also, studies on extended mask use, as well as qualitative studies assessing the impact of mask use in children in different settings and activities will have to be undertaken.

In conclusion, the mask evaluated in this study (with or without MF) is safe for use in children 7 to 14 years old with no underlying medical conditions and in the setting of routine daily activities including brisk walking. The endpoint measure of ETCO_2_ documented no significant rise and the highest ETCO_2_ reading recorded for all subjects was 42 mmHg. It was observed that wearing a mask only marginally increased the ETCO_2_ and FICO_2_ when at rest and with brisk walking. The MF was observed to effectively bring the FICO_2_ levels to a level comparable to that of not wearing a mask.

The current study demonstrates that the mask under study is suitably fit for use in children and the reported comfort level is likely reflective of a good acceptance of its use in the study population.

## Data Availability

The datasets generated and/or analysed during the current study are not publicly available due to confidentiality reasons but are available from the corresponding author on reasonable request.
